# Biopsy-proven ocular sarcoidosis manifesting as Weerfordt-Waldenström syndrome presenting with bilateral anterior granulomatous uveitis, multifocal retinal granulomas, and multifocal choroiditis

**DOI:** 10.1186/s12348-025-00464-y

**Published:** 2025-02-16

**Authors:** Zina Berbich, Imen Ksiaa, Yassir Imani, Sanaa Ahbeddou, Hicham Harmouche, Omar Berbich, Moncef Khairallah

**Affiliations:** 1 Department of Ophthalmology B, Hôpital des spécialités de Rabat, Rabat, Morocco; 2 Department of Ophthalmology, Fattouma Bourguiba University Hospital, Faculty of Medicine, University of Monastir, Monastir, Tunisia; 3 Ophthalmoclinic Nour, Rabat, Morocco; 4 Department of Internal Medicine, Hôpital Ibn Sina, Université Mohammed V de Rabat, Rabat, Morocco

## Background

Sarcoidosis is a systemic granulomatous disease characterized by the formation of epithelioid and giant cell granulomas without caseous necrosis. Diagnosis relies on evidence of systemic granulomatous involvement affecting at least two organs, supported by clinical, laboratory, and histopathological findings. The most affected organs are the mediastinal lymphatic system, lungs, skin, and eyes. Ocular involvement in sarcoidosis occurs among 20 to 50% of patients, and commonly includes adnexal granulomatous infiltration and uveitis [1]. The latter typically manifests with anterior granulomatous uveitis, intermediate uveitis with snowballs, retinal vasculitis or peripheral multifocal choroiditis [1–4]. Posterior segment granulomas are uncommon, and may involve the choroid, the optic nerve, or more rarely the retina [1, 5]. Diagnosis of ocular sarcoidosis is challenging, often made without the gold standard histological proof, based on clinical findings and laboratory results [5]. 

We herein describe a case of biopsy-proven sarcoidosis manifesting as Weerfordt-Waldenström syndrome presenting with anterior granulomatous uveitis, multifocal retinal granulomas, and multifocal choroiditis documented with multimodal imaging including SD-OCT, OCT angiography, and fluorescein angiography.

## Case report

**Fig. 1:**
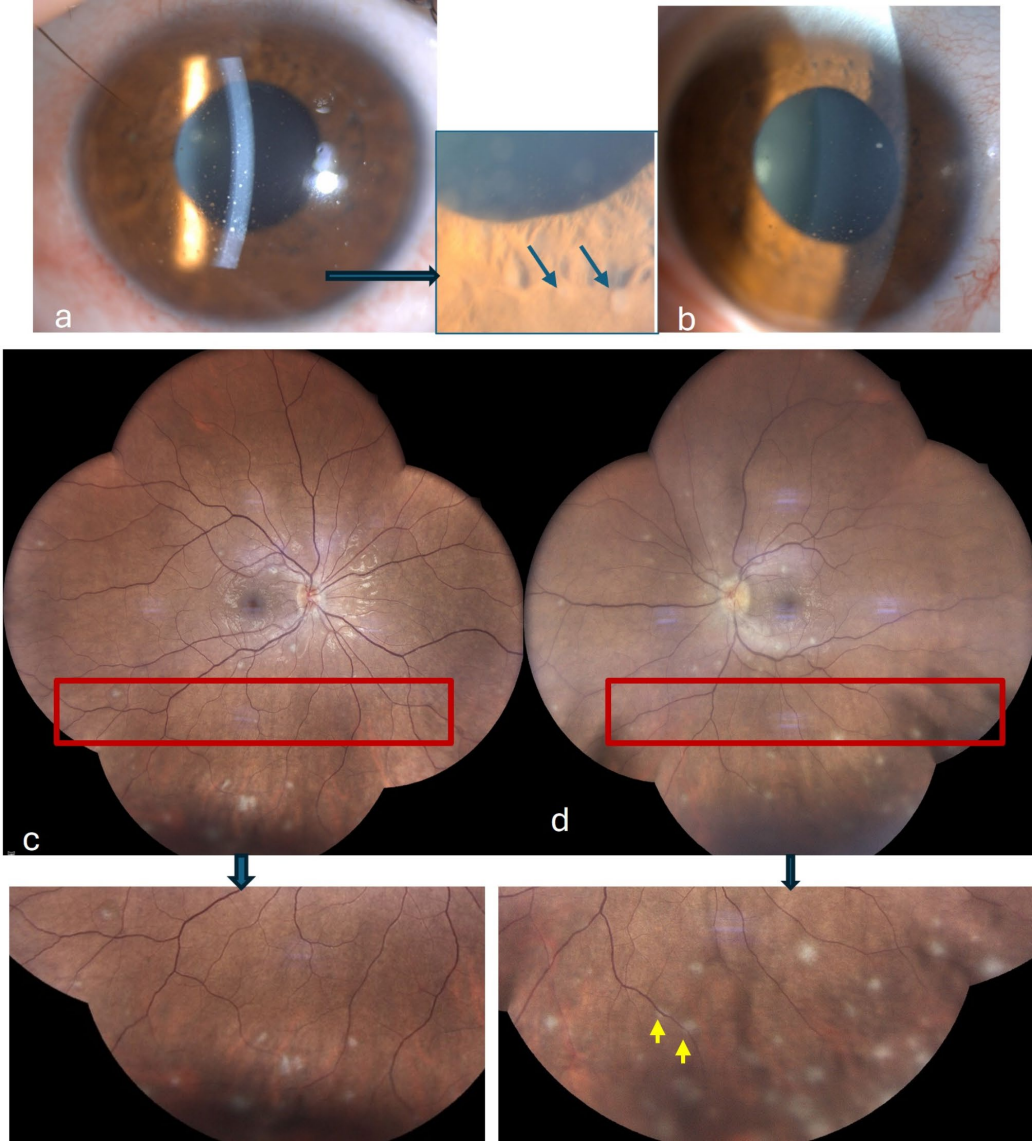
Clinical findings at presentation. (**a, b**) Slit-lamp photographs showing mutton-fat keratic precipitates, and iris nodules in the right (**a**) and left (**b**) eyes better visible in the magnified rectangle. Fundus photographs showing bilateral multifocal and diffuse small whitish lesions located in the posterior pole and inferior retinal periphery (magnified rectangles) in both eyes (**c, d**). Some lesions, mainly in the posterior pole, appeared superficial, while some inferior lesions appeared deeply located, suggesting choroiditis, along with perivascular exudates (yellow arrows)

**Fig. 2:**
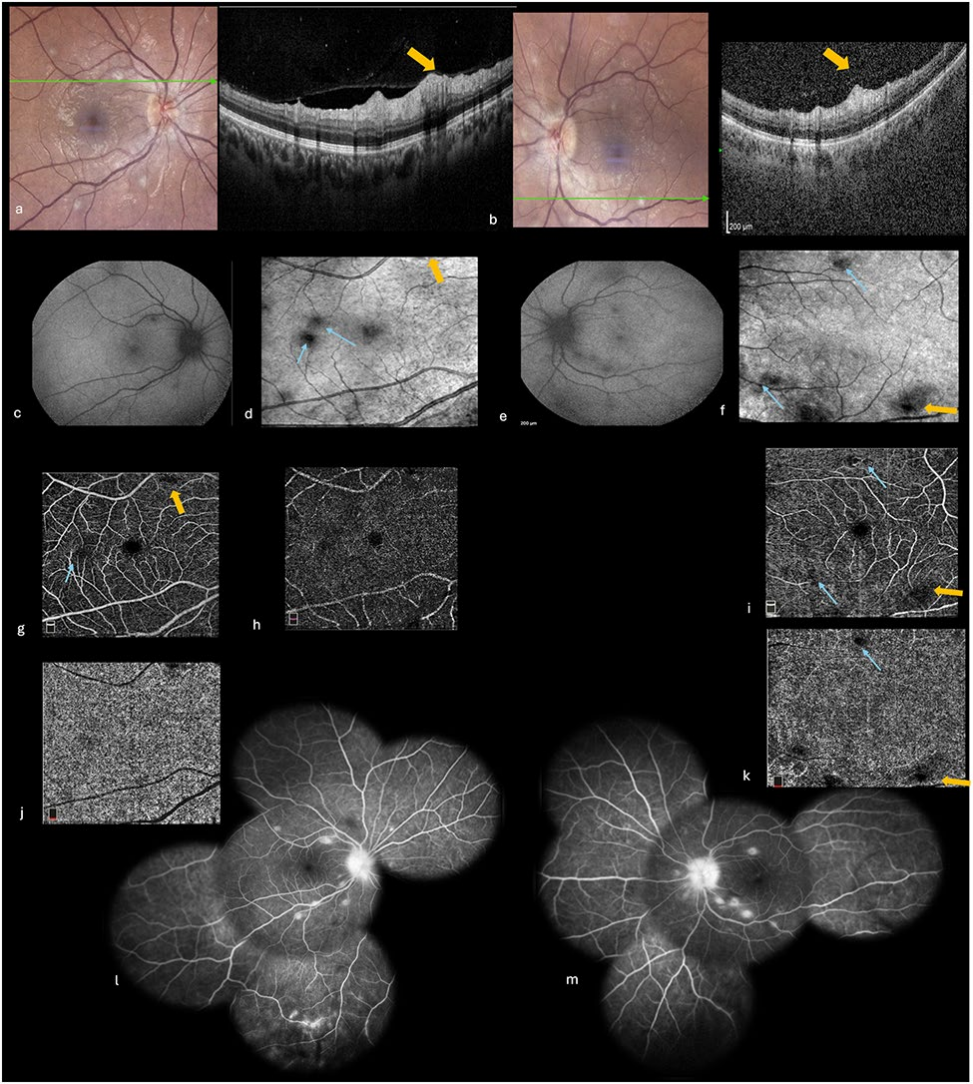
Multimodal imaging at presentation. (**a, b**) SD-OCT scans in the right (**a**) and left (**b**) eyes through posterior pole lesions demonstrating hyperreflective nodular lesions (yellow arrow) at the level of the inner nuclear layer, with sparing of the outer retina, retinal pigment epithelium, and choroid. Fundus autofluorescence photographs showing bilateral multiple hypoautofluorescent lesions in the posterior pole (**c** and **e**), better seen on infra-red images (blue arrows) (**d** and **f**). 6 × 6 mm OCT angiograms showing multifocal areas of no-detectable flow signal in the retinal vascular plexuses and choriocapillaris of the right (**g**, **h** and **j**) and left (**i**, **k**) eyes (blue arrows). Fluorescein angiography showing early hypofluorescence and late staining of chorioretinal lesions, peripheral retinal vascular leakage, and late optic disc hyperfluorescence

A 30-year-old woman with an unremarkable medical history presented with a three-week history of painless bilateral visual loss, fatigue, headaches, cough, night sweats, and fever. On examination, her best-corrected visual acuity (BCVA) was 20/100 in the right eye (RE) and 20/60 in the left eye (LE). There was no relative afferent pupillary defect in either eye. Slit-lamp examination revealed numerous greasy-white mutton-fat keratic precipitates, 2 + cells in anterior chamber, Busacca iris nodules, and 1 + vitreous cells in both eyes. Intraocular pressure was 12 mmHg bilaterally. Fundus examination showed multifocal circular yellow‑white lesions of variable size disseminated throughout the fundus of each eye, mainly in the posterior pole and inferior periphery **(**Fig. 1**)**. The pattern of peripheral lesions was considered to be consistent with classic active sarcoid multifocal choroiditis. Nevertheless, most lesions involving the posterior pole seemed to be located within retina. SD-OCT highlighted in their location hyperreflective nodular lesions primarily involving the inner retinal layers, with sparing of the retinal pigment epithelium and choroid **(**Fig. 2, a **and b)**. Fundus autofluorescence showed hypoautofluorescent spots corresponding to retino-choroidal lesions **(**Fig. 2, c **and e)**. Infra-red images exhibited more lesions than those appreciated by autofluorescence **(**Fig. 2, d **and f)**. OCT angiography showed multifocal areas of no-detectable flow signal in the superficial and deep retinal plexuses and in the choriocapillaris **(**Fig. 2, g-k**)**. Fluorescein angiography showed early hypofluorescence and late staining of all fundus lesions, peripheral retinal vascular leakage, and optic disc hyperfluorescence **(**Fig. 2, l **and m)**.

General physical examination revealed facial nerve palsy and fever. Results of work-up including blood count cells, sedimentation rate, C reactive protein, serologies for rickettsiosis, coxsackie, toxoplasmosis, syphilis, and bartonella, and interferon-gamma release assay were all normal or negative. Results of neurological examination was unremarkable except for facial nerve palsy and fever. A lumbar puncture was performed, and cerebrospinal fluid analysis showed normal formula, negative microbiological analysis, and normal intrathecal angiotensin-converting enzyme (ACE) levels. Results of brain MRI were unremarkable, and serum ACE levels, along with serum protein electrophoresis, were within normal limits. Thoracic computed tomography (CT) imaging demonstrated a non-compressive mediastinal lymphadenopathy. A mediastinal lymph node biopsy revealed non-caseating granulomas composed of epithelioid cells and Langhans giant cells. Therefore, the patient was diagnosed with Weerfordt-Waldenström syndrome, and was treated with topical corticosteroid and intravenous methylprednisolone (10 mg/kg/day) for three days, followed by oral prednisone at 1 mg/kg/day, followed by a gradual tapering schedule for a total period of 12 months.

**Fig. 3:**
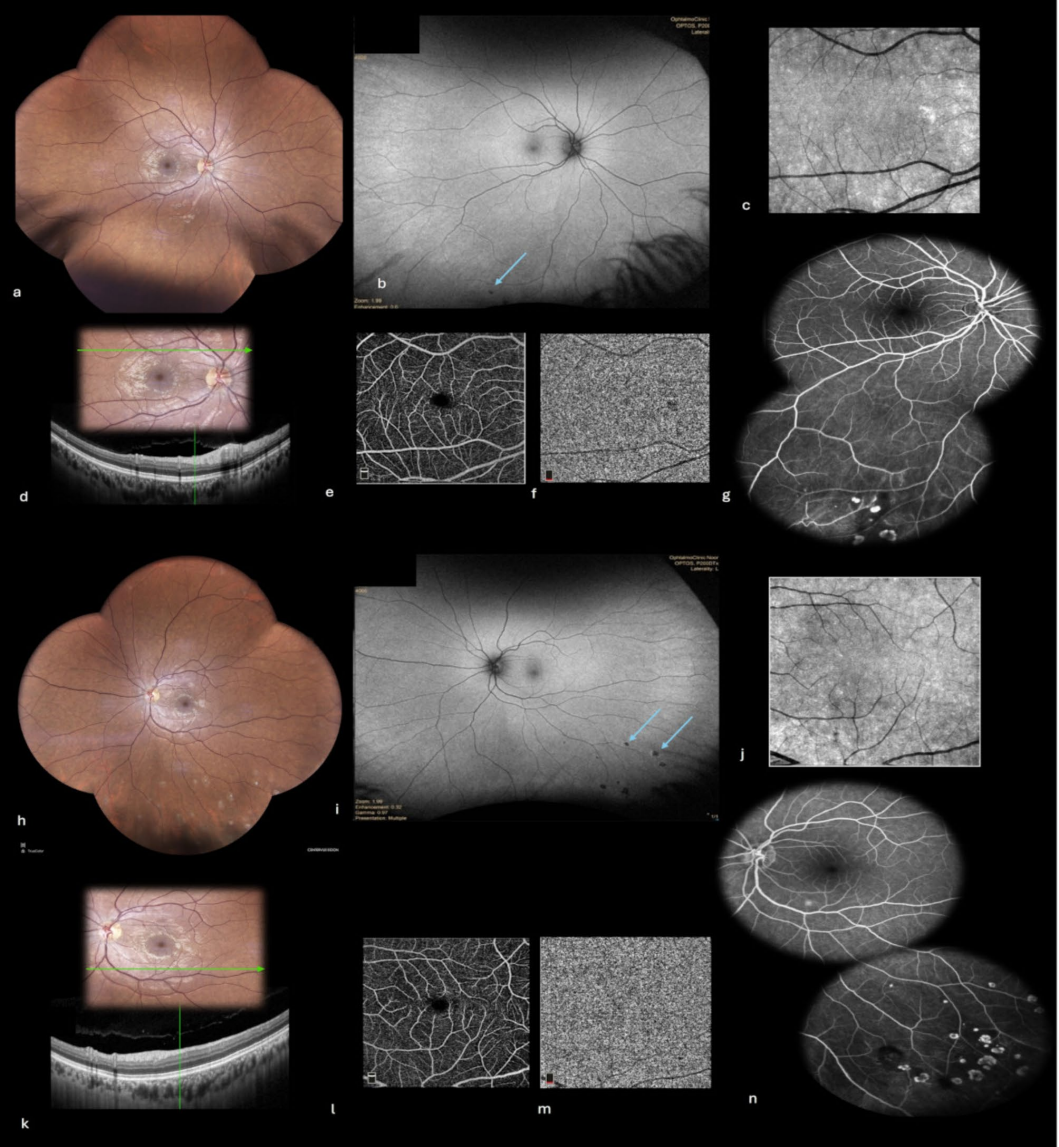
Twelve-month follow-up multimodal imaging (**a-n**). (**a, h**) Fundus photographs showing resolution of most retinal lesions in the posterior pole without leaving residual atrophic scars and the presence of inactive multifocal chorioretinal lesions in the inferior periphery, better illustrated on fundus autofluorescence (**b, i**) (arrows). (**c, j**) Infra-red images showing the resolution of posterior pole retinal lesions. (**d, k**) SD-OCT showing the resolution of retinal hyperreflective lesions, with restoration of retinal layers structure and normalization of flow signal in the superficial capillary plexus on OCT angiography (**e** and **l**), and choriocapillaris (**f** and **m**). Fluorescein angiography exhibiting early hyperfluorescence by window effect of inferotemporal atrophic chorioretinal lesions in both eyes (**g** and **h**)

All systemic symptoms resolved within a few days. Anterior chamber and vitreous inflammation cleared, and visual acuity improved to 20/20 in both eyes. At twelve-month follow-up, all retinal granulomas had completely resolved without leaving residual chorioretinal scars on clinical examination, autofluorescence, or fluorescein angiography. SD-OCT showed the complete restoration of retinal structures at the level of these resolved granulomas. Conversely, several fundus lesions located inferiorly at the acute stage had evolved into areas of chorioretinal atrophy, consistent with a diagnosis of inactive multifocal choroiditis **(**Fig. 3**)**.

## Discussion

In this report, we describe a unique case of biopsy-proven sarcoidosis manifesting with Weerfordt-Waldenström syndrome presenting with bilateral anterior granulomatous uveitis and a combination of multifocal retinal granuloma-like formation and classic multifocal choroiditis. Delineation of chorioretinal involvement at presentation and its evolution under corticosteroid treatment were documented with multimodal imaging including SD-OCT, autofluorescence, OCTA, and fluorescein angiography.

Posterior segment sarcoid granulomas are uncommon and are typically found in the choroid or optic nerve [1–3]. Retinal granulomas are even more unusual and have been reported only in a few patients [6–8]. They have typically been described as large, solitary, elevated vascularized retinal lesions, usually located at the superior or inferior temporal vascular arcade. SD-OCT has been shown to be useful in demonstrating isolated retinal granuloma formation without choroidal involvement and in identifying morphological changes including disorganized retinal architecture, retinal thickening, serous retinal detachment, and posterior hyaloid thickening [6, 7]. 

Consistent with previous reports, our data indicate that sarcoid retinal involvement may also present with multiple small intraretinal nodules sparing the choroid. While the anatomic localization of granulomas may be made clinically, this rarely reported type of sarcoid retinal lesions may be overlooked or confused with the classic chorioretinal lesions, usually seen in ocular sarcoidosis. Fluorescein angiography may show early hypofluorescence and late staining whether inflammatory lesions are retinal or choroidal in origin. Our results, consistent with previous data, show that SD-OCT offers a highly useful objective, noninvasive method of detecting multifocal sarcoid retinal granulomas, assessing lesion depth, and excluding associated choroidal involvement. Hyperreflective elevated nodular lesions involving inner retinal layers similar to those seen in our patient have been previously described. Other OCT patterns of sarcoid retinal granulomas have been described including small intraretinal hyperreflective lesions, preretinal extension, deep retinal layer involvement, and associated retinal pigment and choroidal involvement [6, 7]. 

In our patient, treatment with corticosteroids led to rapid improvement in visual acuity and resolution of acute inflammatory changes. Follow-up examination allowed retinal granulomas to be further distinguishable from chorioretinal lesions. Retinal granulomas completely resolved without leading to residual atrophic chorioretinal scars on clinical examination, autofluorescence, or fluorescein angiography. It is also noteworthy that normal retinal structure completely restored at the level of the resolved retinal granulomas. Conversely, some peripheral active lesions evolved into areas of cobblestone-like chorioretinal atrophy, which was consistent with typical sarcoid multifocal choroiditis. OCT imaging through peripheral lesions was not performed in our patient at presentation. This would have helped to better discriminate between retinal and choroidal involvement by peripheral sarcoid lesions. It is widely assumed that choroidal involvement is more common than retinal involvement in patients with ocular sarcoidosis, and this might be explained by greater vascularization and fenestration zones in the choroid, facilitating immune interactions [8]. Furthermore, the similarity of the blood-retinal and the blood-brain barriers, acting as an effective neuro-immune protector, might explain the rare occurrence of sarcoid granulomatous inflammation in the retina as well as in the neurological system. It is noteworthy that up to a third of patients with neuro-sarcoidosis have ocular involvement including retinal periphlebitis, cotton-wool spots and optic disc swelling [9]. Conversely, neurosarcoidosis has been reported in most patients presenting with retinal granulomas. Neurological manifestations included headaches, recurrent epileptic seizures, granulomatous leptomeningitis, and cerebellar ataxia.

## Conclusion

This case illustrates a unique clinical presentation of definite ocular sarcoidosis in the setting of Weerfordt-Waldenström syndrome confirmed by transbronchial lung biopsy. The patient presented with bilateral granulomatous anterior uveitis associated with multifocal superficial retinal granulomas and multifocal choroiditis. The use of SD-OCT allowed for a precise characterization of the retinal lesions, providing valuable insights into retinal granulomas and their evolution under systemic treatment. Additional studies in ocular sarcoidosis using wide-field OCT scans through peripheral lesions may better characterize and analyze retinal granulomas without or with associated choroidal involvement that could be more common than previously thought.

## Data Availability

No datasets were generated or analysed during the current study.

## References

[1] Rosenbaum JT, Pasadhika S, Ocular, Sarcoidosis (2024) Clin Chest Med 45(1):59–70. 10.1016/j.ccm.2023.08.003 38245371 10.1016/j.ccm.2023.08.003

[2] Jamilloux Y, Kodjikian L, Broussolle C, Sève P (2014) Sarcoidosis and Uveitis. Autoimmun Rev 13(8):840–849. 10.1016/j.autrev.2014.04.001 24704868 10.1016/j.autrev.2014.04.001

[3] Niederer RL, Sharief L, Tomkins-Netzer O, Lightman SL (2023) Uveitis in Sarcoidosis - Clinical features and comparison with other non-infectious Uveitis. Ocul Immunol Inflamm 31(2):367–373. 10.1080/09273948.2022.2032189 35201961 10.1080/09273948.2022.2032189

[4] Garweg JG (2017) [Sarcoidosis and uveitis: an update]. Ophthalmol Z Dtsch Ophthalmol Ges 114(6):525–533. 10.1007/s00347-016-0405-7 10.1007/s00347-016-0405-7 27904945

[5] Mochizuki M, Smith JR, Takase H, Kaburaki T, Acharya NR, Rao NA, International Workshop on Ocular Sarcoidosis Study Group (2019). Revised Criteria of International Workshop on Ocular Sarcoidosis (IWOS) for the Diagnosis of Ocular Sarcoidosis. Br. J. Ophthalmol 103(10):1418–1422. 10.1136/bjophthalmol-2018-313356 10.1136/bjophthalmol-2018-313356 30798264

[6] Wong M, Janowicz M, Tessler HH, Goldstein DA (2009) High-resolution Optical Coherence Tomography of presumed sarcoid retinal granulomas. Retina Phila Pa 29(10):1545–1546. 10.1097/IAE.0b013e3181bd2ffc 10.1097/IAE.0b013e3181bd2ffc 19898191

[7] Gunzinger JM, Fasler K, Al-Sheikh M, Stahel M, Zweifel S (2023) Optical coherence tomography of Retinal Granulomas in presumed ocular sarcoidosis. Klin Monatsbl Augenheilkd 240(4):563–565. 10.1055/a-2009-0667 37164437 10.1055/a-2009-0667

[8] de Saint Sauveur G, Gratiot C, Debieb AC, Monnet D, Brézin AP (2022) Retinal and pre-retinal nodules: a rare manifestation of probable ocular sarcoidosis. Am J Ophthalmol Case Rep 26:101525. 10.1016/j.ajoc.2022.101525 35464687 10.1016/j.ajoc.2022.101525 PMC9020101

[9] Sarac E, Erzurum SA, Arif A (2022) An unusual presentation of Neurosarcoidosis. Am J Case Rep 23:e937125. 10.12659/AJCR.937125 36164269 10.12659/AJCR.937125 PMC9527853

